# ‘Boxing in the corner’: A modified retrograde approach for the management of proximal ureteric stones of 1–2 cm

**DOI:** 10.1080/2090598X.2021.1881421

**Published:** 2021-02-02

**Authors:** Omar Farid Elgebaly, Hussein Abdeldaeim, Tamer Abouyoussif, Ahmed Mahmoud Fahmy, Faisal Edris, Abdelrahman Zahran, Akram Assem

**Affiliations:** Department of Urology, Faculty of Medicine, Alexandria University, Alexandria, Egypt

**Keywords:** Lithotripsy, laser, proximal ureter, stones, ureteroscopy

## Abstract

**Objectives**: To study a modification to the conventional retrograde ureteroscopic approach for treating proximal ureteric stones of 1–2 cm; we intentionally push the stone from the proximal ureter into a favourable calyx then the flexible ureteroscope is used to fragment the trapped stone using laser lithotripsy (‘boxing in the corner’).

**Patients and methods**: The study was conducted in a randomised prospective manner and included 100 patients who presented with a single proximal ureteric stone of 1–2 cm. We randomised the patients into two equal groups: Group A (50 patients) underwent the conventional retrograde technique (CRT) and Group B (50 patients) underwent the modified retrograde technique (MRT) with the primary intention of relocating the stone into a favourable calyx. Intended relocation of the proximal ureteric stone in the MRT group was achieved in a stepwise manner. All intraoperative parameters and postoperative outcomes were recorded and compared between the two groups.

**Results**: There was no statistical significant difference in terms of the patients’ demographics and stone criteria between the two groups. The stone-free rate (SFR) was significantly higher in Group B (92%) compared to Group A (78%) (*P* = 0.049). Fluoroscopy time was significantly longer in Group B (*P* < 0.001), while operative time, lithotripsy time and hospital stay were comparable. There was no difference between the groups regarding complications.

**Conclusion**: The MRT was found to be safe and more effective than the CRT for treating proximal ureteric stones of 1–2 cm, with a significantly higher SFR.

**Abbreviations** CONSORT: Consolidated Standards of Reporting Trials; ESWL: extracorporeal shockwave lithotripsy; fURS: flexible ureteroscope; NCCT: non-contrast CT; SFR: stone-free rate; YAG: yttrium-aluminium-garnet

## Introduction

The optimum treatment for proximal ureteric stones of 1–2 cm remains controversial. Extracorporeal shockwave lithotripsy (ESWL) is associated with a lower stone-free rate (SFR) for stone sizes of >1 cm in the early follow-up period up to 1 month and it is associated with more re-treatments and secondary procedures [1]. A retrograde approach with *in situ* stone lithotripsy is considered the most popular technique used; however, auxiliary interventions are usually required [1,2]. An antegrade approach achieves higher SFRs; however, its adverse effects should be considered [3,4]. Different studies have compared retrograde and antegrade approaches for treating large proximal ureteric stones and the outcome usually refers to a better SFR with the antegrade approach reaching up to 100%, but with a higher incidence of complications regarding the puncture [5,6]. The main reason for the lower SFR in the retrograde approach is the accidental upward migration of large fragments during the *in situ* lithotripsy; these fragments disperse in different calyces due to the irrigant fluid and/or the retropulsive effect of the lithotripsy modality [7]. These fragments will appear later in the postoperative radiology hindering the SFR and may require auxiliary procedures. Modification of the retrograde approach is essential to benefit from its safety and to enhance its SFR. The aim of the present research was to study a modification to the retrograde approach by intentionally manipulating the proximal ureteric stone and pushing it up into a favourable calyx, then the trapped stone is fragmented using a holmium-yttrium-aluminium-garnet (YAG) laser. We refer to fragmentation of the trapped stone after being intentionally pushed upwards inside a calyx as ‘boxing in the corner’. Using this modification, we try to avoid the main cause for lowering the SFR of the retrograde approach, where the stone is fragmented in a closed space with no chance to escape. Although this technique may actually be implemented by many endourologists, to our knowledge it has not been studied specifically and compared to the conventional retrograde approach.

## Patients and methods

### Patient allocation

The present study was conducted between January 2018 and October 2019 after approval of the Ethics Committee of Alexandria University. Inclusion criteria were adult patients aged >18 years and who presented with a single proximal ureteric stone of 1–2 cm. We excluded pregnant females, large stones of >2 cm, concomitant renal stones >0.5 cm, radiolucent stones, and pre-stented patients. All patients enrolled in the present study signed an informed consent.

### Preoperative evaluation and randomisation

All patients underwent full laboratory investigations and non-contrast CT (NCCT) of the abdomen and pelvis for assessment of the stone criteria. The patients were randomised into two groups: Group A (50 patients) underwent the conventional retrograde technique (CRT), and Group B (50 patients) underwent the modified retrograde technique (MRT). Randomisation was done before the operation by a nurse using a closed envelope method.

### Study endpoints

The primary endpoint in this study was the SFR at 4 weeks, which was defined as the absence of fragments of >0.2 cm on NCCT. Secondary endpoints included operative time, lithotripsy time, fluoroscopy time, and need for auxiliary procedures. Operative time was calculated from the beginning of cystoscopy until JJ stent insertion. In our institution JJ stent insertion for 4 weeks is considered a routine step when treating a large proximal ureteric stone of >1 cm to avoid stressful emergencies. Intra- and postoperative complications were recorded and classified according to the modified Clavien–Dindo grading system.

### Interventions

All surgeries were performed by two experienced endourologists and all the data were recorded in a prospective manner

#### Group A: Conventional retrograde technique (CRT)

All patients received general anaesthesia and were placed in the lithotomy position. Cystoscopy was initially done to identify the ureteric orifice and two hydrophilic tipped guidewires (Sensor PTFE-Nitinol guidewire with hydrophilic tip; Boston Scientific Corp., Natick, MA, USA) were inserted. One wire was used as a safety wire, while the other wire was used to backload a flexible ureteroscope (fURS; Flex X2, Karl Storz Endoscope, Tuttlingen, Germany), which was advanced over the wire till reaching the stone. Intracorporeal lithotripsy was done by holmium-YAG laser (Auriga XL 50-W holmium laser, Boston Scientific Corp.) using a 200-µm fibre. The laser energy was applied at a setting of 0.5–0.8 J/pulse and frequency of 20 Hz with short pulse duration to achieve stone lithotripsy. Saline was used as the irrigant fluid and manual pressure was sometimes applied by a syringe to obtain clear vision. After complete dusting of the stone the surgeon had to inspect the renal calyces systematically for any sizable residual stone fragment, which was treated by laser lithotripsy. A JJ stent was inserted in all patients at the end of the procedure (5 F, 26 cm, Percuflex; Boston Scientific Corp.).

#### Group B: Modified retrograde technique (MRT)

All patients received general anaesthesia. Intended migration of the proximal ureteric stone into a renal calyx was achieved in a stepwise manner according to the protocol agreed by the two attending surgeons.

1. Patients were tilted with head down at 30° with the table inclined to the opposite side of the stone so that the upper calyx is always at a lower level than other calyces giving a greater chance for the upper calyx to receive the stone after being intentionally pushed up.
2. After localisation of the stone with fluoroscopy, cystoscopy was done with localisation of the ureteric orifice. A hydrophilic tipped guidewire (Sensor PTFE-Nitinol guidewire with hydrophilic tip, Boston Scientific Corp.) was then used to negotiate the stone trying to relocate it inside the kidney.
3. A ureteric catheter was passed over the guidewire just below the stone, and a jet of saline was injected under manual pressure by a syringe trying to push the stone upwards.
4. A 6-F semi-rigid URS (Karl Storz Endoscope) was used to pass inside the ureter till reaching the stone and irrigation fluid was injected under pressure.
5. If the stone is hardly impacted and not responding to the previous steps, the laser was used cautiously to fragment the periphery of the stone trying to disimpact the stone from the ureteric wall while preserving the whole bulk of the stone to be fragmented in the kidney after its upward migration.

After the upward stone migration was successfully achieved using the manoeuvres mentioned above in a stepwise manner, the fURS was advanced over the guidewire till reaching the kidney. Stones migrated to the renal pelvis were manipulated with the fURS and irrigant fluid till reaching a favourable calyx. If the stone migrated into an unfavourable calyx, a tipless nitinol basket was used to relocate the stone in the upper calyx. Stones entrapped in the calyx were dusted using the fURS and holmium-YAG laser using the same machine and settings as in the CRT group. A JJ stent (5 F, 26 cm, Percuflex from Boston Scientific Corp.) was inserted at the end of all procedures and removed after 4 weeks.

### Statistical analysis

Data were analysed using the IBM Statistical Package for the Social Sciences (SPSS®) software package, version 20.0 (IBM Corp., Armonk, NY, USA). The Kolmogorov–Smirnov, Shapiro and D’Agstino tests were used to verify the normality of distribution of variables. Comparisons between groups for categorical variables were assessed using chi-square test (Fisher’s or Monte Carlo). The Student’s *t*-test was used to compare the two groups for normally distributed quantitative variables. The Mann–Whitney test was used to compare between the two groups for abnormally distributed quantitative variables. Significance of the obtained results was judged at the 5% level. The power of the study was calculated using the Power and Sample Size program, version 3.0, January 2009 (available at: https://ps-power-and-sample-size-calculation.software.informer.com/3.0/).

## Results

**Figure 1.**
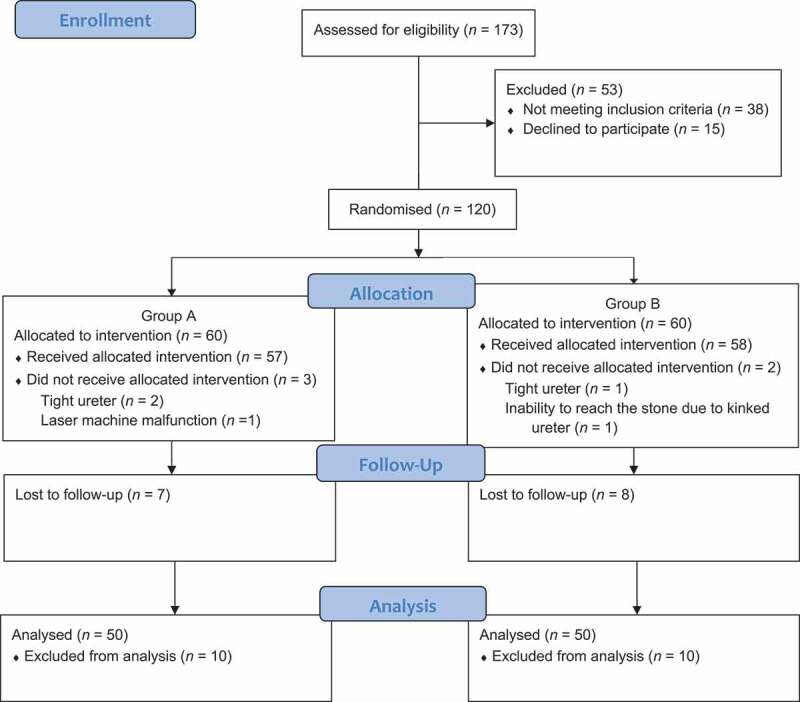
CONSORT flowchart of the study

From January 2018 to October 2019, 173 patients were enrolled in the study and tested for eligibility. The Consolidated Standards of Reporting Trials (CONSORT) flowchart is used to demonstrate the flow of the enrolled patients in the study (Figure 1). A total of 53 patients did not meet the inclusion criteria and were excluded; the remaining 120 patients were randomised between both groups. Five patients failed to continue the intervention as presented in the flowchart and a JJ stent was inserted. After exclusion of 15 patients who were lost to follow-up, a total 100 patients (50 patients in each group) were enrolled in the final analysis. Group A underwent the CRT, while Group B underwent the MRT. Both groups were comparable in terms of demographics and stone criteria as shown in Table 1. Intraoperative parameters and postoperative outcomes were recorded and are described in Table 2. The mean (SD) operative time was comparable in groups A and B, at 62 (7.5) and 66.3 (14.9) min, respectively (*P* = 0.072). The mean (SD) lithotripsy time was also comparable in groups A and B, at 28 (6.4) and 29.6 (8.5) min, respectively (*P* = 0.276). The mean (SD) fluoroscopy time was significantly higher in Group B than Group A, at 58.9 (28.4) vs 12.5 (3.4) s (*P* < 0.001).

**Table 1.** Patients’ demographics and stone criteria

Variable	Group A, CRT	Group B, MRT	*P*
Number of patients	50	50	
Age, years, mean (SD)	44.5 (11.8)	44.1 (11.1)	0.876
Sex, *n* (%)			
Male	23 (46)	26 (52)	0.548
Female	27 (54)	24 (48)	
Stone criteria			
Size, cm, mean (SD)	1.46 (0.2)	1.39 (0.18)	0.102
Density, HU, mean (SD)	897.8 (175.9)	964.6 (214.2)	0.092
Laterality, *n* (%)			
Right	23 (46)	26 (52)	0.548
Left	27 (54)	24 (48)	
Hydronephrosis, *n* (%)			
No	12 (24)	10 (20)	0.933
Grade I	20 (40)	23 (46)	
Grade II	12 (24)	11 (22)	
Grade III	6 (12)	6 (12)

**Table 2.** Intraoperative variables and postoperative outcomes

Variable	Group A, CRT (*n* = 50)	Group B, MRT (*n* = 50)	*P*
Operative time, min, mean (SD)	62 (7.5)	66.3 (14.9)	0.072
Lithotripsy time, min, mean (SD)	28 (6.4)	29.6 (8.5)	0.276
Fluoroscopy time, s, mean (SD)	12.5 (3.4)	58.9 (28.4)	<0.001*
Residual stone (at 4 weeks), *n* (%)			
No (=SFR)	39 (78)	46 (92)	0.049*
Yes	11 (22)	4 (8)	
Hospital stay, days	1.0	1.0	–
Postoperative complications, *n* (%)			
Haematuria (mild)	13 (26)	15 (30)	0.656
Pain (mild to moderate)	21 (42)	25 (50)	0.422
Bladder irritative symptoms	32 (64)	27 (54)	0.309
Low-grade fever	6 (12)	4 (8)	0.505

*Statistically significantly.

The SFR after 4 weeks was significantly higher in Group B, where 92% had no residual stones in comparison to Group A where only 78% had no residual stones (*P* = 0.049). The mean residual stone size in Group A was 0.46 cm while in Group B was 0.32 cm. Moreover, at 3 months Group B did not need any further urological interventions to achieve a stone-free status because all the residual fragments passed spontaneously, unlike Group A where nine patients needed ESWL and two patients passed their residual fragments spontaneously. As regard postoperative hospital stay, all patients in both groups were discharged within 24 h. According to the Clavien–Dindo Classification for complications, minor complications (Grade I) were recorded in both groups including mild haematuria and low-grade fever, with no major complications reported.

In Group B with the MRT, the mean (SD) time needed for intended migration was 8.6 (3.5) min. This extra time did not add significantly to the total operative time when compared with Group A. Table 3 describes the methods needed to achieve the intended migration. After intended migration was achieved four stones (12.5%) were displaced into an unfavourable calyx and required a tipless nitinol basket to re-position the stone in the upper calyx for stone fragmentation.

**Table 3.** Methods used to achieve intended migration in group B

Method	*N* (%)
Only positioning	0 (0)
Guidewire manipulation	6 (12)
Ureteric stent and forced irrigation	5 (10)
Semi-rigid URS and forced irrigation	20 (40)
Laser fragmentation of the stone periphery	19 (38)

## Discussion

Stone migration during retrograde management of proximal ureteric stones is considered the main reason hindering the SFR and consequently increases the need for auxiliary procedures [8,9]. Stone migration may occur due to the pressurised irrigant, which is mandatory for proper visualisation during stone fragmentation or due to the retropulsive effect of the laser lithotripsy. The upward displaced fragments maybe noticed during the procedure and thus can be managed during the same session; however, it is not uncommon to miss the migrated fragments and discover them during the follow-up. Different techniques have been introduced in an attempt to increase the SFR of proximal ureteric stone management. Anti-retropulsive devices significantly reduce stone migration and this is reflected by higher SFRs; however, they increase the cost of the procedure and add to the risk of complications [7]. Meanwhile, these devices may not be able to pass above the stone due to stone impaction or they may fail to completely secure the dilated ureter above the stone giving space for some fragments to escape between the device and the ureteric wall. Modifications for treating proximal ureteric stones have been reported in many clinical trials achieving superior outcomes and improving the SFR (Table 4 [10–17]).

**Table 4.** Modifications for treating proximal ureteric stones

Reference	Type of modification	Stone size, cm, mean (SD) or mean (range)	Number of patients, CRT/modification	SFR, CRT/modification, *n* (%)	*P*
Pan et al., [10]	Trendelenburg position	1.001 (0.33)	176/179	164 (93.2)/176 (98.3)	0.016
Zhou et al., [11]	Trendelenburg position + stone basket	1.14 (0.33)	96/96	70 (72.9)/85 (88.5)	0.006
Jung et al., [12]	Antegrade irrigation via percutaneous nephrostomy	1.023 (0.434)	89/45	73 (82)/43 (95.6)	0.033
Sfoungaristos et al., [13]	Antegrade ureteroscopy using fURS	2.14 (0.487)	34/23	28 (82.4)/23 (100)	0.033
Elgebaly et al., [14]	Antegrade mini-percutaneous flexible ureteroscopy	1.33 (0.23)	30/30	18 (60)/25 (80)	0.045
Gu et al., [15]	Minimally invasive PCNL using rigid 8.5/9.8-F URS	1.727 (1.5–2.5)	29/30	26 (89.7)/30 (100)	0.07
Chen et al., [16]	Push back the stone via retrograde ureteroscopy and stone retrieval via supine PCNL	2.01 (0.63)	31	30 (96.8)	
Huang et al., [17]	Suctioning flexible ureteroscopy with intelligent pressure control	2.37 (0.43)	10	10 (100)	

PCNL, percutaneous nephrolithotomy.

Treating the stone in the proximal ureter while it is surrounded by inflammatory mucosal folds that hinder proper visualisation of the stone makes the procedure rather tedious. Fragmenting the stone in this position risks injuring the surrounding mucosa or the safety guidewire and at the same time it is more vulnerable to retropulsion. The operator should interrupt the fragmentation more frequently to ensure the laser beam is away from the mucosa and the safety guidewire. The irrigation pressure should be lowered to avoid migration of the stone, especially when the stone gets smaller and less impacted, and this will add to the difficulty of the procedure and may increase the risk of injuring the surrounding mucosa due to the unclear vision [18].

The aim of the present modification is the early control of the inevitable stone migration and to turn this event into an advantage that increases the success of the procedure, meanwhile it allows fragmentation of the stone in a more capacious place with less risk of mucosal injury and away from the safety guidewire. While the stone is trapped in the calyx the procedure can be carried out with more comfort, as the irrigant pressure can be elevated safely allowing clearer vision and the fragmentation will not be interrupted to avoid injuring the mucosa.

The stepwise techniques in the present study insured the successful trapping of the stone in a favourable calyx, thus enabling successful fragmentation of the stone. The time needed to achieve intended stone migration did not add significantly to the total operative time, with both groups recording comparable mean operative times (*P* = 0.072). On the other hand, the need of fluoroscopic tracking of the stone until it reached a favourable calyx in Group B was reflected by a significant increase in the mean fluoroscopy time compared to Group A (*P* < 0.001). The SFR after 4 weeks was statistically significantly higher in Group B, reflecting the superiority of the ‘boxing in the corner’ concept.

From our point of view the quality of stone fragmentation in the MRT group was better than the CRT group. Patients in the MRT group with residual stones were able to pass their residual fragments spontaneously, reflecting proper dusting of the initial stone and good quality of the residual small fragments that did not necessitate any further secondary procedures, they only needed more time to pass out and they disappeared at the 3-month follow-up. On the other hand, most of the patients in the CRT with residual stones were not able to pass their fragments spontaneously and they required further procedures in the form of ESWL, indicating poor quality of large-sized residual fragments.

A limitation of our present series is that it was carried in a single centre. The small number of cases presents another limitation. Although this modified technique may be adopted by many surgeons on an individual basis, to our knowledge there is no publication in the literature that has studied this procedure or compared it to the conventional technique. In our opinion, further prospective multicentre studies with a larger number of cases may propagate this technique aiming to improve results.

## Conclusion

In conclusion, ‘boxing in the corner’ using the MRT showed comparable safety and higher efficacy than the CRT in treating proximal ureteric stones of 1–2 cm.
